# Preparation of Waterborne Silicone-Modified Polyurethane Nanofibers and the Effect of Crosslinking Agents on Physical Properties

**DOI:** 10.3390/polym16111500

**Published:** 2024-05-24

**Authors:** Fang Li, Kai Weng, Asumi Nakamura, Keishiro Ono, Toshihisa Tanaka, Daisuke Noda, Masaki Tanaka, Shinji Irifune, Hiromasa Sato

**Affiliations:** 1 Interdisciplinary Graduate School of Science and Technology, Shinshu University, 3-15-1, Tokida, Ueda-Shi 386-8567, Nagano, Japan; 20hs155a@shinshu-u.ac.jp (F.L.); 20hs152f@shinshu-u.ac.jp (K.W.);; 2 Silicone-Electronics Materials Research Center, Shin-Etsu Chemical Co., Ltd., 1-10, Hitomi, Matsuida-Machi, Annaka-Shi 379-0224, Gunma, Japan; 3 Dainichiseika Color & Chemicals Mfg. Co., Ltd., 2087-4, Ohta, Sakura-Shi 285-0808, Chiba, Japan

**Keywords:** waterborne silicone-modified polyurethane, electrospinning, nanofiber membrane, crosslinking agent, heat treatment

## Abstract

Silicone-modified polyurethane (PUSX) refers to the introduction of a silicone short chain into the polyurethane chain to make it have the dual properties of silicone and polyurethane (PU). It can be used in many fields, such as coatings, films, molding products, adhesives, and so on. The use of organic solvents to achieve the fiberization of silicone-modified polyurethane has been reported. However, it is challenging to achieve the fiberization of silicone-modified polyurethane based on an environmentally friendly water solvent. Herein, we report a simple and powerful strategy to fabricate environmentally friendly waterborne silicone-modified polyurethane nanofiber membranes through the addition of polyethylene glycol (PEG) with different molecular weights using electrospinning technology and in situ doping with three crosslinking agents with different functional groups (a polyoxazoline crosslinking agent, a polycarbodiimide crosslinking agent, and a polyisocyanate crosslinking agent) combined with various heating treatment conditions. The influence of PEG molecular weight on fiber formation was explored. The morphology, structure, water resistance, and mechanical properties were analyzed regarding the effect of the introduction of silicone into PU. The effects of the type and content of crosslinking agent on the morphology and physical properties of PUSX nanofiber membranes are discussed. These results show that the introduction of silicone can improve the water resistance and high temperature resistance of waterborne PU, and the addition of a crosslinking agent can further improve the water resistance of the sample, so that the sample can maintain good morphology after immersion. Crosslinking agents with different functional groups had different effects on the mechanical properties of PUSX nanofiber membranes due to different reactions. Among them, the oxazoline crosslinking agent had a significant effect on improving tensile strength, while the isocyanate crosslinking agent had a significant effect on improving the elongation at break. The PUSX nanofiber membrane prepared in this work did not use organic solvents that were harmful to humans and the environment, and it can be used in outdoor textiles, oil–water separation, medical health, and other fields.

## 1. Introduction

Waterborne polyurethane resin (WPU) is a green polyurethane system that uses water instead of an organic solvent as the dispersion medium. It has the advantages of being non-toxic and pollution-free, good compatibility, and easy modification [1,2]. However, due to the hydrophilic group and linear structure of the waterborne polyurethane chain, its water resistance, solvent resistance, and mechanical properties are inferior to the organic solvent polyurethane [3,4], which limits its wider application. The incorporation of silicone functional groups is one of the effective methods to improve the water resistance and hydrophobicity of waterborne polyurethane [5,6,7,8]. The main chain Si-O-Si of the silicone compound belongs to the “inorganic structure”, while the side chain Si-CH_3_ belongs to the “organic structure”. Its unique chemical structure makes it possess the dual advantages of inorganic and organic compounds, such as a low glass transition temperature, hydrophobicity, oxidation resistance, low surface energy, good thermal stability, and water resistance [9,10]. Its excellent performance has attracted extensive attention from researchers [11,12].

Wu et al. [13] prepared a flexible polyurethane foam composite with stable conductivity and high flame retardancy by immersing RGO-coated PU foam into a solution containing silicone resin and silica nanoparticles and curing it. Dai et al. [14] used PDMS-modified waterborne polyurethane as a polymer matrix to prepare a conductive coating to improve the water resistance and surface hydrophobicity of coated textiles and applied it to electromagnetic shielding materials. These silicone-containing polyurethane materials are mainly used as films, molding products, or adhesives. On the other hand, there are few reports of silicone-containing PU in the form of nanofibers. Electrospinning is one of the most common methods to prepare continuous nanofibers. Nanofibers prepared using electrospinning technology have the advantages of small size, large specific surface area, and high porosity. Based on these superior properties, a variety of polymer nanofibers have been widely studied and electrospinning is used to manufacture various materials [15]. Gu et al. [16] coated PDMS on PU/PCL nanofibers to obtain composite nanofiber membranes, showing a water contact angle of 141°, with good waterproof and breathable properties. However, the poor durability of the coating affects its application performance. Zhu et al. [17] used electrospinning technology to prepare PMHS/PU nanofiber membranes with water-resistant and breathable properties using N, N-dimethylacetamide (DMAc) and isopropanol (IPA) as solvents. Gu et al. [18] prepared polyurethane/silica dioxide (PU/SiO_2_) waterproof and breathable nanofiber membranes through electrospinning and hydrothermal treatment. However, these toxic solvents are not eco-friendly and are harmful to humans and the environment. In our previous studies, electrospinning silicone-modified polyurethane nanofibers were prepared using organic solvents [10,19]. As far as we know, nanofibers based on waterborne silicone-modified polymers have not been reported.

In this study, we prepared waterborne silicone-modified polyurethane (PUSX) by introducing silicone groups into the polyurethane backbone and tried to make it achieve fiberization through electrospinning. The existence of entanglement between polymer chains is a necessary condition for the preparation of nanofibers using electrospinning technology. Typically, high molecular weight polyethylene glycol (PEG) is used as the template polymer for preparing waterborne polyurethane nanofibers, imparting excellent flexibility and elasticity to the fibers. Additionally, PEG itself possesses outstanding water solubility, biocompatibility, and degradability. Moreover, PEG can increase the viscosity of the spinning solution and promote molecular chain entanglement, thereby facilitating fiber formation [20,21,22]. However, it is difficult to directly prepare nanofibers via electrospinning using only the waterborne PUSX dispersion due to its low viscosity, insufficient molecular chain entanglements, and unfavorable factors caused by silicone modification, such as the decreased polymer chain polarity of PU, increased electrical insulation, and changes in surface tension. To achieve emulsion electrospinning, high molecular weight water-soluble template polymers are usually required to provide viscoelasticity and aid the formation of fibers. Therefore, we tried to perform the fiberization of waterborne PUSX through the addition of PEG with different molecular weights and different proportions to waterborne PUSX dispersion. However, due to the hydrophilic groups in the molecular chain of PEG and waterborne PUSX, the prepared PUSX nanofibers have poor water resistance and solvent resistance, and it is difficult to maintain the fiber morphology properly after water immersion. The defect of the poor water resistance of waterborne polyurethane is usually improved by adding a crosslinking agent. The commonly used waterborne polyurethane crosslinking agents mainly include a polyisocyanate crosslinking agent, an aziridine crosslinking agent, a polycarbodiimide crosslinking agent, etc. [23,24,25,26]. Typically, the crosslinking reaction not only enhances water resistance but also improves the mechanical properties of the material [27]. Due to the presence of hydrophilic groups (carboxyl groups) similar to those in waterborne polyurethane, in waterborne silicone-modified polyurethane, this study aimed to enhance the performance of waterborne PUSX nanofiber membranes. Various types of crosslinking agents (such as carbodiimide crosslinking agents, oxazoline crosslinking agents, and isocyanate crosslinking agents) were added to investigate the effects of crosslinking agent type, content, and crosslinking conditions on the morphology, water resistance, and mechanical properties of waterborne PUSX/PEG nanofiber membranes systematically.

## 2. Materials and Methods

### 2.1. Materials

Waterborne silicone-modified polyurethane (PUSX), polyurethane (PU), and crosslinking agents were provided by Shin-Etsu Chemical Industry Co., Ltd. (Tokyo, Japan) and Dainichiseika Color & Chemicals Mfg. Co., Ltd. (Tokyo, Japan). Polyethylene glycol (PEG) (Mw = 20,000, 100,000, 500,000 g/mol) was provided by FUJIFILM Wako Pure Chemical Co., Ltd. (Tokyo, Japan). The solid content of both PUSX and PU solution was 30 wt%. In addition, the concentration of silicone was 10 wt% and the length of the silicon chain was 20. The crosslinking agents (PyC) used were a polycarbodiimide crosslinking agent (PCC), polyoxazoline crosslinking agent (POC), and polyisocyanate crosslinking agent (PIC). In the later description, the y of the crosslinking agent PyC stands for C, O, and I. Table 1 shows the parameters of the crosslinking agent.

### 2.2. Synthesis of Waterborne PUSX and PU

**Figure 1:**
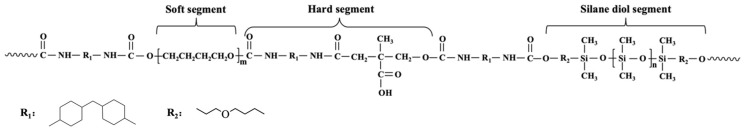
The molecular structures of waterborne silicone-modified polyurethane.

Two-terminal silicone diols (Figure S1(1-1)) were prepared from Si-H group-containing organopolysiloxanes and unsaturated alcohols catalyzed by a platinum catalyst [10]. The prepolymer of terminal isocyanate was synthesized in acetone solution by using polytetramethylene diol (Poly THF 2000), 4,4-methylenebis (cyclohexyl isocyanate) (H12MDI), the two-terminal organosilicon diol prepared above, and the water dispersant dihydroxymethylpropionic acid (DMPA) as raw materials. And the chains were extended by adding isophorone diamine (IPDA). Finally, acetone was removed from the mixture via distillation under reduced pressure to obtain an aqueous dispersion of silicone-modified polyurethane resin with a solid concentration of 30 wt%, as shown in Figure S1(1-2). The molecular structure formula of PUSX is shown in Figure 1. This PUSX dispersion is anionic self-emulsifying waterborne polyurethane. The waterborne PU dispersion of 30 wt% was prepared in the same way by changing the amount of material.

### 2.3. Preparation of Waterborne PUSX and PU Nanofibrous Membranes

The spinning solution was obtained by mixing PEG, PUSX/PU, and crosslinking agents at the determined conditions. The concentration and conditions of the crosslinking agent are shown in Table 2. The content of the crosslinking agent is relative to PUSX. The crosslinking agents were added to the spinning solution 8–12 h before electrospinning and stirred thoroughly. Waterborne PUSX/PU nanofiber membranes were prepared using an NEU Nanofiber Electrospinning Unit (Kato Tech Co., Ltd., Kyoto, Japan). The spinning solution was loaded into a 10 mL plastic syringe with a metal needle (18 G, 1.2 mm) and pumped out at a constant rate of 0.15 mm/min. The needle was applied with high voltage maintained at 13–20 kV and the spinning distance was 18 cm to generate continuous PUSX/PU nanofibers. The spinning time was 6–8 h. During the spinning process, the temperature and relative humidity were controlled at 23 ± 2 °C and 50%, respectively.

### 2.4. Characterization

#### 2.4.1. Scanning Electron Microscopy

The surface morphology of nanofibers at an accelerating voltage of 10 kV was determined using a scanning electron microscope (JSM-6010LA JEOL, Tokyo, Japan). The samples were coated with platinum layers using a platinum sputter coater (Ion Sputter JFC-1600 JEOL Ltd., Tokyo, Japan) at 30 mA with a sputtering time of 80 s. The fiber diameters were measured using Image J 1.5.3e software. At least 50 fibers were analyzed for each sample, and the average diameter of electrospinning nanofibers was calculated.

#### 2.4.2. Chemical Structure Analysis by FTIR

Attenuated total reflection–Fourier transform infrared spectroscopy (FT/IR-6600 IRT-5200, JASCO, Hachioji, Japan) was used to analyze the chemical structure of the different samples. The spectra were recorded in the range of 4000–400 cm^−1^ with 64 scans and a resolution of 4 cm^−1^.

#### 2.4.3. Water Performance Comprehensive Evaluation Test

Multiple testing methods were adopted for evaluating water performance. Firstly, we used an automatic contact angle meter (DMS-400, Kyowa Interface Science Co., Ltd., Niiza, Japan) to drop 2 µL of distilled water onto the surface of the sample to measure its static water contact angle. Secondly, we cut the sample into 10 × 10 mm size and soaked it in distilled water at room temperature for 24 h to ensure full saturation of the sample. Subsequently, excess surface water was removed, and the water absorption of the sample was determined by calculating the ratio of the mass difference before and after immersion to the mass before immersion. Finally, after drying the sample at room temperature for 24 h, its water resistance was evaluated by measuring the weight and area change rate before and after immersion. This series of tests collectively revealed the performance characteristics of samples in aqueous environments. The area of the nanofiber membrane was manually measured and calculated using a ruler. All samples were measured three times, and the average value was taken.

The rate of change of weight and area before and after water immersion was calculated by the following equation.

$$m=\frac{m_{2}-m_{1}}{m_{1}}\times 100\%$$

where *m*: rate of change, *m*_1_: original weight of the sample, and *m*_2_: weight of the sample after absorbing distilled water or fully dried.

#### 2.4.4. Porosity Measurements

The porosity of the nanofiber membrane was measured using a liquid displacement method [28]. Hexane was used as the displacement liquid. Because hexane is a non-solvent of waterborne PUSX and easily permeates through interconnected pores, it will not cause shrinkage or swelling. The porosity of the nanofiber membrane was calculated using the following formula:

$$porosity=\frac{V_{1}-V_{3}}{V_{2}-V_{3}}\times 100\%$$

where *V*_1_ and *V*_2_ represent the volume of hexane before and after immersion of the nanofiber membrane, respectively, and *V*_3_ represents the volume of hexane after removal of the membrane.

#### 2.4.5. Tensile Tests

Tensile tests were performed on different samples using a small bench-top universal tensile tester (EZTest/EZ-S, Shimadzu, Kyoto, Japan). The samples with thicknesses ranging from 0.05–0.2 mm were cut to a size of 30 × 5 mm and fixed on a mold, and the samples were fixed on the tensile machine using a fixture. The initial distance between the two fixtures was 10 mm and the crosshead speed was 10 mm/min. The tensile strength, Young’s modulus, and elongation at break were calculated for each sample based on the stress–strain curve. Each sample was tested 10 times under the same conditions and the average value was taken.

#### 2.4.6. Statistical Analysis

The significance of water resistance and physical properties were statistically analyzed via one-way analysis of variance (ANOVA) using GraphPad Prism 9 software [29]. Statistical significance was set at * *p* < 0.05, ** *p* < 0.0001 to identify which groups were significantly different from the other groups.

## 3. Results and Discussion

### 3.1. Fabrication of Waterborne PUSX and PU Nanofiber Membranes

**Figure 2:**
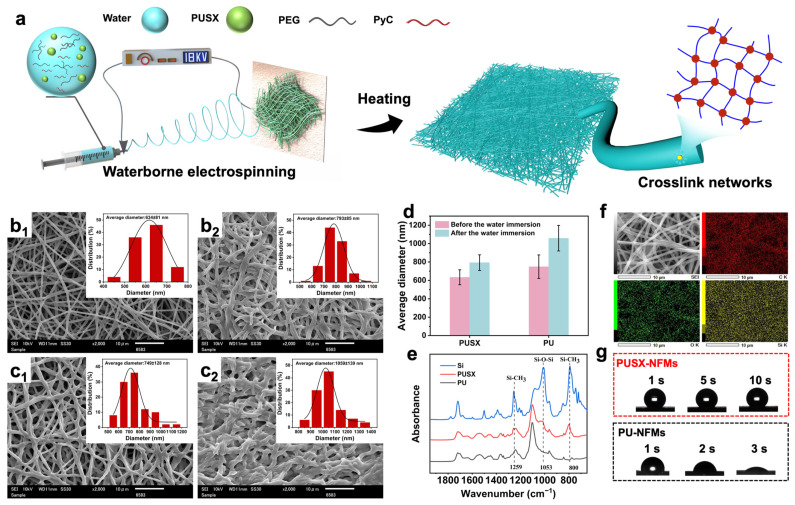
(**a**) Schematic diagram of the fabrication of PUSX nanofiber membranes. (**b_1_**,**c_1_**,**b_2_**,**c_2_**) are waterborne PUSX and PU nanofiber membranes before and after immersion, respectively (The inset shows the diameter distribution.). (**d**) Fiber diameter of waterborne PUSX and PU nanofibers before and after water immersion. (**e**) FTIR spectra of silicone, waterborne PU, and PUSX nanofibers. (**f**) The EDS mapping images of PUSX nanofibers. (**g**) The dynamic photograph of a water droplet (2 µL) remaining on the surface of PUSX and PU membranes.

Generally, the molecular chains of water-based dispersions are short, and the nanofibers cannot be electrospun due to the lack of chain entanglement in the dispersions. The preparation of waterborne polyurethane (WPU) nanofibers using polyvinyl alcohol, polyvinylpyrrolidone, or polyethylene oxide as template polymers has been widely reported [22,30,31]. In this study, the suitable conditions for the fiberization of waterborne PUSX were explored by adding polyethylene glycol (PEG) with different contents and different molecular weights. Here, molecular weights of 20,000, 100,000, and 500,000 PEG were selected, and the optimum fiber-forming conditions were determined by exploring the different ratios of PEG and PUSX. Detailed exploration conditions are shown in Supplementary Discussion 1. Finally, the molecular weight of 500,000, PEG:PUSX = 10:90 conditions were selected for exploration in the following experiment. It was found that the addition of PEG as a template polymer, as well as WPU, enabled the fabrication of PUSX nanofibers without the influence of silicone modification during electrospinning. The schematic diagram of the preparation of PUSX nanofiber membranes (PUSX-NFMs) is shown in Figure 2a. Green and environmentally friendly waterborne PUSX-NFMs were prepared by using a waterborne electrospinning process, adding water-soluble polymer PEG, and in situ doping with a water-based resin crosslinking agent (PyC). After simple heat treatment, a highly interconnected crosslinked network was obtained, and the enrichment of silane groups on the fiber surface was achieved, endowing PUSX-NFMs with good hydrophobicity and water resistance. To explore the successful synthesis of silicone and polyurethane and the improvement of polyurethane performance through the introduction of silicone, waterborne polyurethane nanofibers (PU-NFMs) obtained using the same method were also compared.

### 3.2. Morphological and Structure Characterization of PU and PUSX Nanofibers

As shown in Figure 2(b_1_,c_1_), the SEM images of waterborne PUSX and PU nanofiber membranes without crosslinking agents are shown, respectively. The silicone-modified PUSX nanofibers showed a clear cylindrical structure with an average diameter of 634 nm, while the waterborne PU nanofibers had a larger average diameter (749 nm) due to mutual adhesion. Figure 2(b_2_,c_2_) displays the PUSX and PU fiber membranes after water immersion. After water immersion, the average diameter of PUSX fibers increased to 793 nm (an increase of 25.1%) and the diameter of PU fibers increased to 1059 nm (an increase of 41.4%). It is worth noting that the blurred morphology of PU nanofibers after water immersion may be attributed to the inherent hydrophilicity of waterborne PU and the swelling and dissolution of PEG in water [32]. In contrast, the PUSX nanofibers could still maintain a fibrous structure with less swelling and adhesion after water immersion, indicating that silicone modification endowed the waterborne PU with finer fiber diameters, better water resistance, and narrower diameter distributions (Figure 2(b_1_,c_1_,b_2_,c_2_) insets and Figure 2d). The chemical structure of the membrane was analyzed using FTIR spectroscopy as shown in Figure 2e. In the spectra of PUSX-NFMs, we observed the Si-C stretching vibration peaks at 800 cm^−1^ and 1259 cm^−1^, as well as the Si-O-Si stretching vibration peak at 1053 cm^−1^. The peaks co-occurring in the spectra of PUSX and PU nanofibers are located at 1719 cm^−1^ and 1542 cm^−1^, which correspond to the C=O stretching vibration peak and N-H bending vibration peak of the carbamate group, respectively [10,33]. In addition, the energy dispersive spectroscopy (EDS) images of PUSX nanofibers (Figure 2f) showed that the silicon elements were uniformly distributed on the surface. These results indicate that the silicone molecular chains were successfully added to the polyurethane molecular chains without affecting the fiberization process of polyurethane.

Furthermore, to verify the thermal stability of waterborne PUSX, we observed fiber morphological changes after heating at different temperatures for 24 h, as shown in Figure S5. As the temperature increased, the unmodified PU nanofibers gradually began to adhere to each other, and many fusions between the fibers appeared when the temperature reached 80 °C. This may be caused by the low thermal stability of waterborne PU and the melting of PEG at high temperatures. When the temperature reached 100 °C, cracks appeared on the fibers and the diameter increased significantly. In contrast, the PUSX nanofiber membrane could still maintain a cylindrical shape at high temperatures with a small increase in fiber diameter, demonstrating good heat resistance. In addition, hydrophobic performance was evaluated using the water contact angle test, as shown in Figure 2g. The dynamic water contact angle test showed that the PUSX membrane had excellent hydrophobicity, which was significantly better than the water-based PU membrane.

### 3.3. Optimization of the Crosslinking Conditions of Different Crosslinking Agents

**Figure 3:**
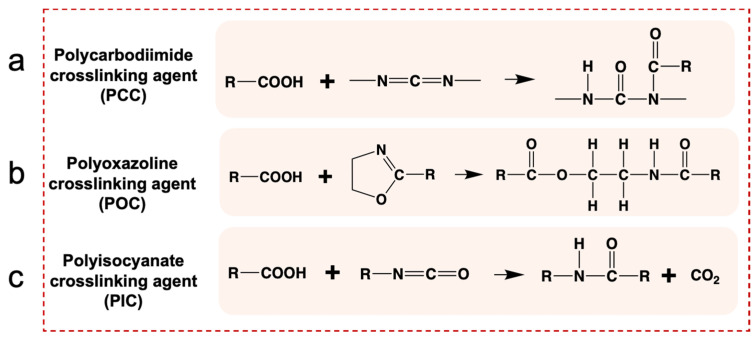
The possible chemical crosslinking reactions between the carboxyl groups (-COOH) in the waterborne PUSX/PU and (**a**) the polycarbodiimide crosslinking agent, (**b**) polyoxazoline crosslinking agent, and (**c**) polyisocyanate crosslinking agent.

To explore the crosslinking conditions of different crosslinking agents, the optimal crosslinking conditions were selected by characterizing the morphology, water resistance, and hydrophobicity of PUSX nanofiber membranes at the crosslinking agent content of 15%. The possible chemical crosslinking reactions between the carboxyl groups (-COOH) in the waterborne PUSX/PU and the polycarbodiimide crosslinking agent (PCC), polyoxazoline crosslinking agent (PCC), and polyisocyanate crosslinking agent (PIC) are illustrated in Figure 3.

**Figure 4:**
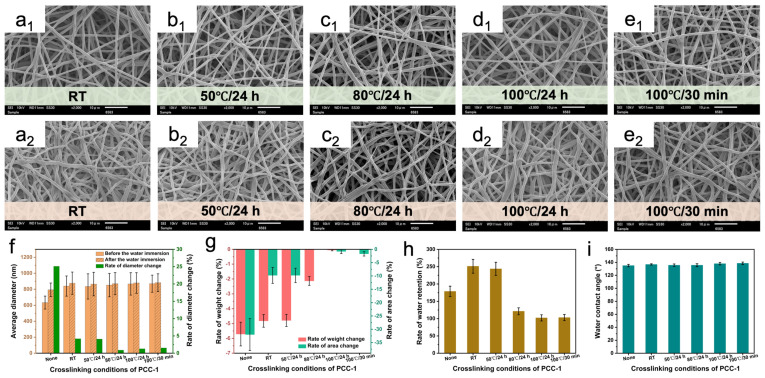
SEM images of PUSX nanofibers before (**a_1_**,**b_1_**,**c_1_**,**d_1_**,**e_1_**) and after (**a_2_**,**b_2_**,**c_2_**,**d_2_**,**e_2_**) water immersion at 15% PCC-1 content. The crosslinking conditions were (**a**) room temperature, (**b**) 50 °C/24 h, (**c**) 80 °C/24 h, (**d**) 100 °C/24 h, and (**e**) 100 °C/30 min. (**f**) Average diameter and diameter change rate, (**g**) weight and area change rate, (**h**) water retention, and (**i**) water contact angle of PUSX nanofibers at 15% PCC-1 content.

Figure 4a–e shows the morphology of PUSX nanofibers with the PCC-1 crosslinking agent before and after immersion in water under different crosslinking conditions. The addition of the crosslinking agent enabled the fibers to maintain good morphological stability after water immersion, without obvious adhesion and swelling. This is mainly due to the reaction between the carbodiimide group and the carboxyl group to generate a stable three-dimensional network structure, which increases the water resistance of the fiber membrane. With the increase in the crosslinking temperature, the nanofibers showed a decreasing trend in diameter change rate, weight, area change rate, and water retention rate before and after water immersion (Figure 4f–h). When the temperature reached 100 °C, the results of crosslinking for 24 h and crosslinking for 30 min were not significantly different, indicating that the high temperature shortened the crosslinking reaction time. The stretching vibration peak of -N=C=N- attributed to PCC at 2110 cm^−1^ in the infrared spectrum gradually decreased with increasing temperature, indicating that more -N=C=N- groups in the crosslinking agent cross-linked with the carboxyl groups in the PUSX nanofibers to generate N-acyl urea groups (-NHCO-NH-CO-) (Figure S6a). The water contact angle (WCA) in Figure 4i showed that the contact angle on the nanofiber surface did not change significantly under different crosslinking conditions. This suggests that the PCC-1 crosslinker may be able to undergo a partial crosslinking reaction at room temperature. It also indicates that the hydrophobic behavior of PUSX nanofibers is mainly determined by the presence of silicone groups rather than the effect of crosslinking temperature. Considering the time and energy consumption factors, we chose 100 °C/30 min as the optimal crosslinking condition of the PCC-1 crosslinking agent in the subsequent experiments. This temperature and time can not only ensure that the crosslinking reaction is carried out adequately but also save energy by avoiding prolonged high-temperature treatment. Similarly, when exploring the optimal crosslinking conditions for the PCC-2 crosslinking agent at 15% content, we also adopted the process parameter of 100 °C/30 min, as described in Supplementary Discussion 2.

**Figure 5:**
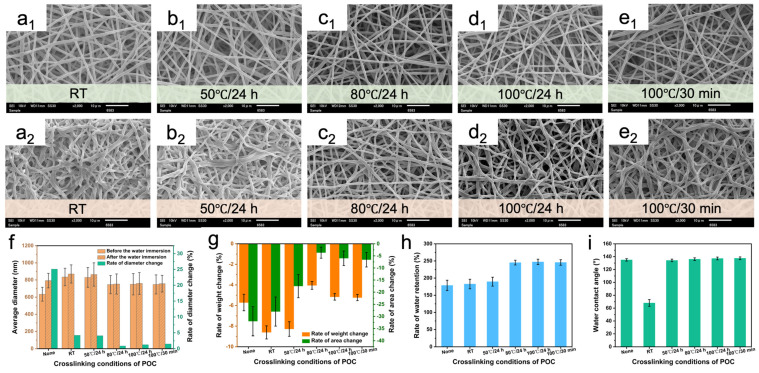
SEM images of PUSX nanofibers before (**a_1_**,**b_1_**,**c_1_**,**d_1_**,**e_1_**) and after (**a_2_**,**b_2_**,**c_2_**,**d_2_**,**e_2_**) water immersion at 15% POC content. The crosslinking conditions were (**a**) room temperature, (**b**) 50 °C/24 h, (**c**) 80 °C/24 h, (**d**) 100 °C/24 h, and (**e**) 100 °C/30 min. (**f**) Average diameter and diameter change rate, (**g**) weight and area change rate, (**h**) water retention, and (**i**) water contact angle of PUSX nanofibers at 15% POC content.

Figure 5a–e shows the morphology of PUSX nanofibers with the POC crosslinking agent before and after immersion in water under different crosslinking conditions. With the increase in the crosslinking temperature, the nanofiber morphology after water immersion was maintained and the diameter change rate (Figure 5f) decreased gradually. The addition of the crosslinking agent resulted in a significant decrease in the rate of weight change and area change (Figure 5g) of the nanofiber membranes. This is due to the opening of the double bond of the oxazoline ring and the crosslinking reaction with the carboxyl group (Figure 3b). After the crosslinking reaction, the significant increase in water resistance of the crosslinked nanofibers could be attributed to the reduction in hydrophilic groups (-COOH) and the elongation of molecular chains. However, as the crosslinking temperature continued to rise to 100 °C, the change in water resistance gradually stabilized, which might be due to the complete inclusion of all hydrophilic groups in the crosslinking reaction. When the crosslinking temperature was below 80 °C, the diameter and rate of change of the nanofibers before and after immersion were relatively large, which may be due to it not reaching the reaction temperature of the oxazoline group crosslinking agent. Alternatively, hydrophilic PEG undergoes swelling or dissolution under the action of water, leading to an increase in fiber diameter. As the crosslinking temperature increased, the crosslinking reaction occurred, and the number of crosslinking points gradually increased, forming a stable interconnected porous network structure in the fiber membrane, which improved water resistance. Furthermore, the water retention ability of the nanofiber membrane also improved as the temperature increased (Figure 5h). This is because the POC crosslinking agent cannot undergo a crosslinking reaction with PEG (for detailed information, see Supplementary Discussion 4), resulting in water absorption and swelling of the PEG. 

Figure 5i shows the water contact angle of the samples. It can be observed that the unheated sample has a smaller water contact angle and poor hydrophobicity. This may be due to the addition of a POC crosslinking agent while introducing too much water into the spinning solution, resulting in a decrease in silane concentration. Moreover, the oxazoline ring is a heterocyclic structure containing nitrogen atoms, with polarity and hydrophilicity, which may be one of the reasons for the decrease in hydrophobicity. Alternatively, crosslinking reactions can only be initiated at a certain temperature, and high temperatures can promote the enrichment of hydrophobic silane on the fiber surface, thereby increasing hydrophobicity. The FTIR spectra of PUSX/POC-NFMs are shown in Figure S6b. The -C=N- characteristic peak appeared at 1651 cm^−1^ and the characteristic peak intensity at 1349 cm^−1^ decreased with increasing temperature. When the temperature reached 80 °C, the characteristic peak almost disappeared, indicating that the crosslinking reaction had occurred completely. Considering the morphology, structure, water resistance, and other factors, the optimal crosslinking condition of 80 °C/24 h was determined and applied for subsequent experiments.

**Figure 6:**
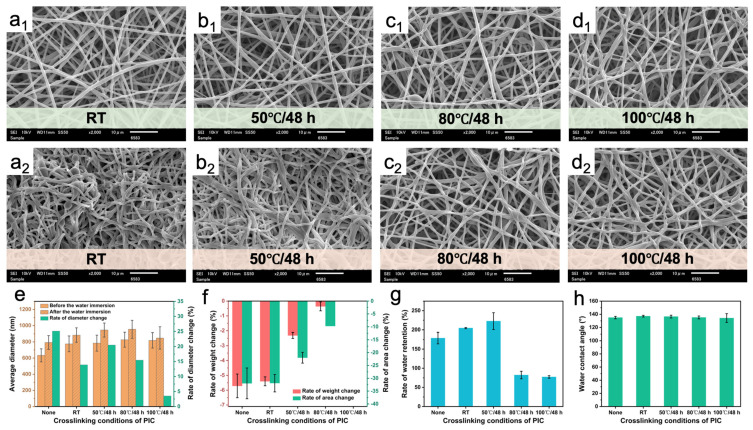
SEM images of PUSX nanofibers before (**a_1_**,**b_1_**,**c_1_**,**d_1_**) and after (**a_2_**,**b_2_**,**c_2_**,**d_2_**) water immersion at 15% PIC content. The crosslinking conditions were (**a**) room temperature, (**b**) 50 °C/48 h, (**c**) 80 °C/48 h, and (**d**) 100 °C/48 h. (**e**) Average diameter and diameter change rate, (**f**) weight and area change rate, (**g**) water retention, and (**h**) water contact angle of PUSX nanofibers at 15% PIC content.

Figure 6a–d shows the morphology of PUSX nanofibers with the PIC crosslinking agent before and after immersion in water under different crosslinking conditions. With similar results to the PCC and POC crosslinking agents, the crosslinking reaction could not occur or occurred slowly when the crosslinking temperature was low. When the crosslinking temperature was lower than 80 °C, the fibers dissolved after water immersion, resulting in larger fiber diameters (Figure 6e). As the crosslinking temperature increased, the rate of change of fiber diameter, weight, and area before and after water immersion significantly decreased (Figure 6f). When the crosslinking temperature reached 100 °C, the shape and weight of the fiber membrane remained stable before and after water immersion. Moreover, the water retention of the PUSX nanofiber membrane crosslinked decreased at high temperatures (Figure 6g) and the water contact angle remained almost constant (Figure 6h), indicating that the occurrence of the crosslinking reaction increased the water resistance of the nanofiber membrane without affecting the hydrophobicity of the fiber surface. Figure S6c shows the FTIR spectra under different crosslinking conditions. The spectrum of PIC showed the characteristic peak of -N=C=O at 2253 cm^−1^. As the crosslinking temperature increased, the stretching vibration peaks belonging to C=O in urethane and the bending vibration peak of N-H in 1696 and 1541 cm^−1^ became stronger, indicating that the crosslinking reaction was more complete at high temperatures. Finally, 100 °C/48 h was selected as the optimal crosslinking condition for the PIC crosslinking agent for subsequent experiments.

### 3.4. Morphology of the PUSX-PyC Nanofiber Membranes

To investigate the effects of different crosslinking agents (PyC) and different contents on PUSX nanofibers, we characterized the morphology, structure, and mechanical properties of the different samples under optimized conditions.

**Figure 7:**
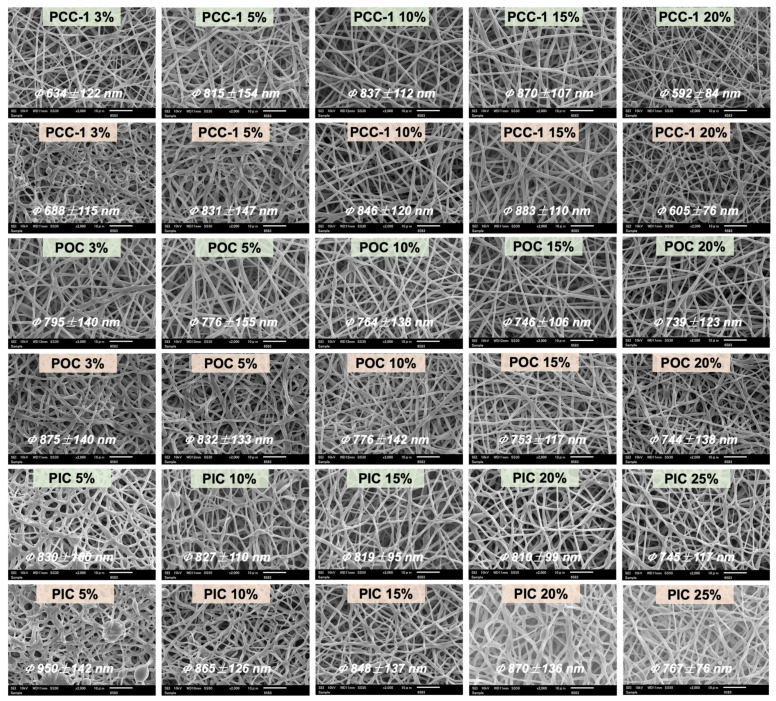
PUSX/PyC-NFM morphology before and after water immersion at different contents PCC-1, POC, and PIC crosslinking agents.

**Figure 8:**
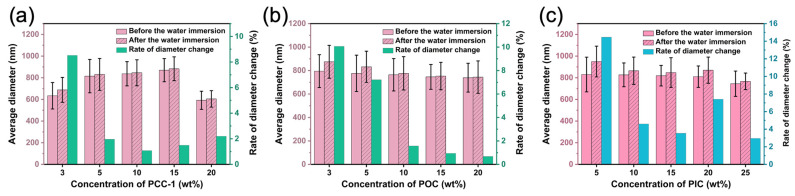
The average fiber diameter and diameter change rate of PUSX nanofibers with (**a**) PCC-1, (**b**) POC, and (**c**) PIC before and after water immersion.

Figure 7 shows the morphology of PUSX/PyC nanofiber membranes (NFMs) before and after water immersion with different contents of PyC crosslinking agents (PCC, POC, PIC). For PCC-1, the fiber morphology after water immersion showed good shape stability with increasing crosslinking agent content, and the fiber diameter change rate (Figure 8a) was also significantly lower, indicating that the crosslinking agent improved the water resistance of PUSX-NFMs. When the content was 10%, the diameter change rate was reduced to 1.1% compared to the non-added sample (25.1%). When the crosslinking agent content was too high, the rate of change of fiber diameter before and after water immersion showed a small increasing trend, which might be caused by excessive crosslinking leading to adhesion between fibers. And when the crosslinking agent content reached 20%, a large number of bead structures appeared in the fiber morphology, which may be due to the instability of the spinning process caused by the high concentration of the crosslinking agent. With the increase in POC crosslinking agent concentration, the nanofiber diameter remained relatively stable before and after water immersion, suggesting that the crosslinking reaction had reached saturation, resulting in stable water resistance and the complete reaction of hydrophilic groups. However, the decreasing rate of diameter change (Figure 8b) may be attributed to the saturation of crosslinking, where the excess unreacted hydrophilic crosslinking agent dissolved, leading to a smaller diameter change rate before and after water immersion. These findings were consistent with the subsequent discussion on water resistance, water retention, and other related test results. The SEM diagram and diameter change rate of the PIC crosslinking agent before and after water immersion (Figure 8c) showed a similar change to that of the PCC-1 crosslinking agent. With the increase in crosslinking agent content, the fiber morphology after water immersion showed good shape stability, and the diameter change rate showed a decreasing trend. However, an excessive amount of crosslinking agent causes the diameter change rate to show a slightly increasing trend. This may be due to the high concentration of the crosslinking agent leading to instability in the electrospinning process and adhesion between adjacent nanofibers. This showed that the crosslinking reaction generated more crosslinked structures with the increase in crosslinking agent content within a certain range, which increased the water resistance of PUSX/PyC-NFMs. In contrast, the results of the PCC-2 crosslinking agent concentration showed a larger rate of change in fiber diameter after crosslinking, indicating a poor crosslinking effect. Detailed results are shown in Supplementary Discussion 3.

### 3.5. Transform Infrared Spectroscopy Analysis

**Figure 9:**
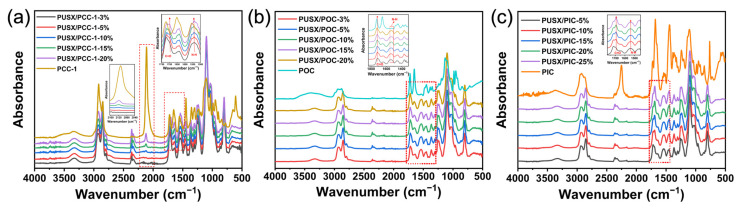
FTIR spectra of PUSX nanofibers with the PyC crosslinking agent at different contents. (**a**) PCC-1, (**b**) POC, and (**c**) PIC.

To investigate the effect of different crosslinking agents and contents on the chemical structure of PUSX nanofiber membranes, FTIR measurements were performed on samples after heat treatment with different conditions as shown in Figure 9. The characteristic peak of the functional group (-N=C=N-) in the PCC-1 crosslinking agent appears at 2110 cm^−1^ as shown in Figure 9a. When the crosslinking reaction occurs, the double bond of the -N=C=N- functional group opens and reacts with -COOH to form the N-acyl urea group (Figure 3a). In the infrared spectrum, enhancement of characteristic peaks belonging to C=O stretching vibration at 1698 cm^−1^ and N-H bending vibration at 1541 cm^−1^ can be observed. When the content of the crosslinking agent exceeded 10%, the characteristic peak of -N=C=N- was still observed in the FTIR spectra. This phenomenon might be attributed to the excessive amount of the crosslinking agent, resulting in a portion of the crosslinking agent remaining in the system without being involved in the crosslinking reaction. Figure 9b shows the FTIR spectra of PUSX nanofibers for the addition of a POC crosslinking agent. The oxazoline group in the POC crosslinking agent reacts with -COOH to form ester-amine groups. It can be seen from the figure that the characteristic peaks belonging to the -C=N functional group of POC appearing at 1651 cm^−1^ with different crosslinking agent concentrations have disappeared, and the characteristic peak belonging to -NHC=O at 1719 cm^−1^ and the characteristic peak of N-H at 1538 cm^−1^ have been enhanced, indicating that the crosslinking reaction has occurred. The FTIR spectrum of the added PIC crosslinking agent is shown in Figure 9c, and the tensile vibration peak of its characteristic functional group -N=C=O appears at 2253 cm^−1^. With the increase in crosslinking agent content, the -N=C=O group reacts with the -COOH group to form the acetamide group. In the infrared spectrum, the characteristic absorption peaks appearing at 1696 cm^−1^ and 1541 cm^−1^ correspond to the C=O stretching vibration and N-H bending vibration, respectively. The intensity of these two peaks gradually increases with the increase in the crosslinking degree.

### 3.6. Water Resistance Evaluation

**Figure 10:**
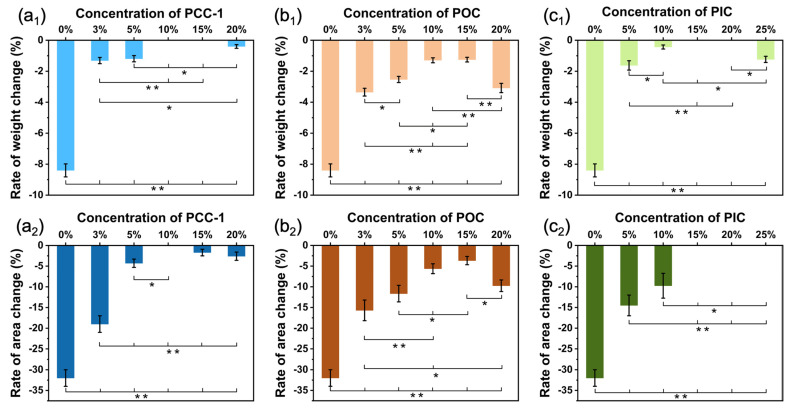
The rate of change in weight (**a_1_**,**b_1_**,**c_1_**) and area (**a_2_**,**b_2_**,**c_2_**) before and after water immersion with the (**a**) PCC-1, (**b**) POC, and (**c**) PIC crosslinking agents. * *p* < 0.05 and ** *p* < 0.0001 are considered to indicate statistically significant differences in the rate of change in weight and area of the nanofiber membranes with different crosslinking agent contents before and after immersion.

To explore the effect of crosslinking agent addition on the water resistance of PUSX nanofiber membranes, water immersion experiments were performed on samples with different crosslinking agents and different crosslinking conditions. The results of sample weight and area change rate before and after water immersion are shown in Figure 10. With the increase in PCC-1 crosslinking agent content, the weight and area change rate before and after water immersion showed a trend of decreasing. The reason for the decrease is that the crosslinking reaction improves the water resistance of the nanofiber membrane. When the content of crosslinking agent is too high, due to the weak interaction forces between the crosslinking agent and polymer molecules (such as hydrogen bonding, van der Waals forces, etc.) to stably bind them, a phase separation phenomenon occurs, forming a crosslinking agent-enriched phase and a polymer-enriched phase. This phase separation behavior may affect the morphology and shape stability of nanofibers, such as the beaded fibers shown in the SEM images.

The same trend of variation is also shown in the POC and PIC crosslinking agents. Under a certain range, as the crosslinking agent content increases, more hydrophilic functional groups (-COOH) in the waterborne PUSX react with the crosslinking agent, making the water resistance of the nanofiber increase and thus the rate of change before and after immersion decrease.

### 3.7. Water Retention Evaluation

**Figure 11:**
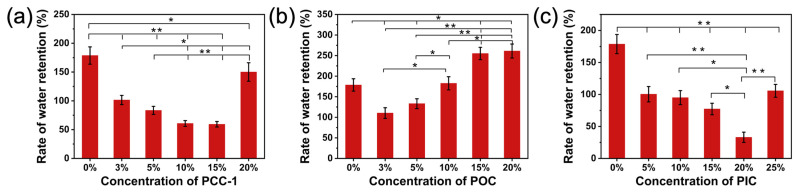
Water retention rate of PUSX nanofibers with PyC at different crosslinking contents: (**a**) PCC-1, (**b**) POC, and (**c**) PIC. * *p* < 0.05 and ** *p* < 0.0001 are considered to indicate statistically significant differences in the water retention rate of the nanofiber membranes with different crosslinking agent contents.

Figure 11 shows the relationship between the water retention and crosslinking agent content of the nanofiber membranes. With the increase in the content of the PCC-1 crosslinking agent (Figure 11a), the water absorption of the nanofiber membrane decreased, that is, the water retention rate decreased. This result showed that the crosslinking reaction improved water resistance. High content of crosslinking agents, including unreacted crosslinking agents, leads to electrospinning instability, and many bead structures appear, which enlarge fiber pores, decrease water resistance, and increase water retention. The water retention of POC showed an increasing trend with the increase in the crosslinking agent content (Figure 11b), which is different from the results of PCC and PIC. This may be attributed to the fact that the oxazoline groups only react with carboxyl groups in PUSX, but not with the hydroxyl groups in PEG. Detailed information is displayed in Supplementary Discussion 4.

A similar trend to PCC-1 was also observed in the case of the PIC crosslinking agent (Figure 11c). A moderate increase in crosslinking agent content can enhance crosslinking density, stabilize the fiber structure, and reduce the number of hydrophilic groups, thereby improving water resistance and decreasing water retention. Nonetheless, when the crosslinking agent content becomes too high, the water retention of all three crosslinking agents increases again, possibly due to the introduction of excessive water or the accumulation of crosslinking agents on or within the fibers, forming a loose porous structure. Therefore, it is crucial to reasonably control the crosslinking agent content, seek a balance between improving water resistance, and avoid excessive water absorption to achieve optimal nanofiber membrane performance.

### 3.8. Water Contact Angle

**Figure 12:**
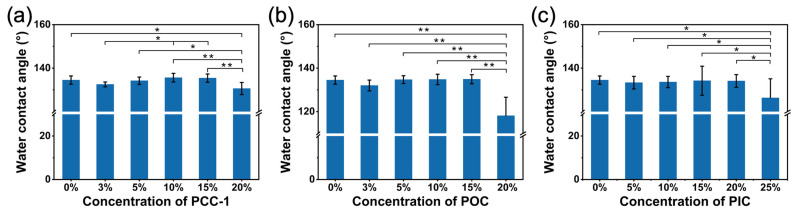
Water contact angle of PUSX nanofibers with PyC at different crosslinking contents: (**a**) PCC-1, (**b**) POC, and (**c**) PIC. * *p* < 0.05 and ** *p* < 0.0001 are considered to have statistical significance between the different contents of crosslinking agents.

To characterize the effects of different crosslinking agents on the hydrophobic properties of PUSX nanofiber membranes, the static water contact angle was measured for different samples as shown in Figure 12. In a certain concentration range, the water contact angle of the nanofiber membranes increased slightly with the increase in PyC content. This result shows that the characteristic functional groups in all three crosslinking agents can react with the -COOH in the aqueous PUSX to crosslink the reaction, and the increase in water resistance leads to a slight increase in surface hydrophobicity. However, overall, all samples showed a large water contact angle, indicating that for the three crosslinking agents and their concentrations, fiber diameter had little effect on the hydrophobicity of PUSX nanofiber membranes. The large influence on the hydrophobic properties is due to the silicone modification contained in the samples. The low surface energy silicone on the nanofiber surface will develop, conferring its hydrophobic properties.

### 3.9. Characterization of Mechanical Properties

**Figure 13:**
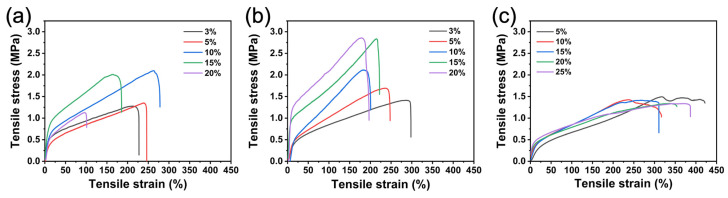
Tensile stress–strain curves of (**a**) PCC-1, (**b**) POC, and (**c**) PIC at different contents.

**Figure 14:**
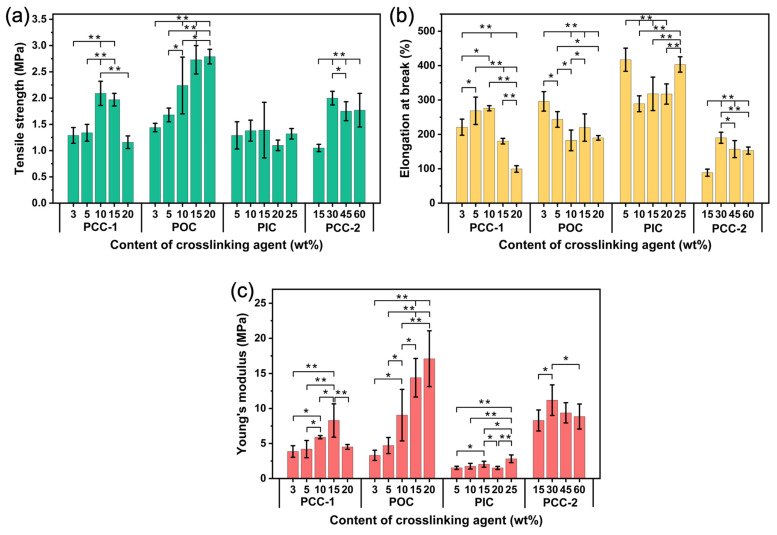
The mechanical properties of PyC-PUSX-NFMs at different crosslinking agent and content: (**a**) tensile strength, (**b**) elongation at break, and (**c**) Young’s modulus. * *p* < 0.05 and ** *p* < 0.0001 are considered to have statistical significance between the different contents of crosslinking agents.

As we know, good mechanical properties are very important in practical applications. To explore the effect of the crosslinking agents on mechanical properties, tensile tests were carried out on the samples. Figure 13 shows the stress–strain curves of different crosslinking agents at different contents. The stress–strain curve of the PUSX nanofiber membrane without a crosslinking agent is shown in Figure S12. As shown in Figure 13 and Figure 14, different crosslinking agents have different effects on mechanical properties.

For the PCC-1 crosslinking agent (Figure 13a), the tensile strength, elongation at break, and Young’s modulus of the nanofiber membranes increased slightly with increasing crosslinking agent concentration at appropriate content. Namely, the tensile strength increased from 1.29 ± 0.15 MPa to 2.09 ± 0.23 MPa, elongation at break increased from 221.0 ± 23.4% to 276.1 ± 7.5%, and Young’s modulus increased from 3.86 ± 0.83 MPa to 5.90 ± 0.23 MPa when the concentration was increased from 3% to 10%. The carbodiimide (-N=C=N-) in the PCC-1 crosslinking agent reacted with the carboxyl group to form the N-acyl urea groups [22]. From the results of the mechanical tests, the moderate N-acyl urea crosslinking point improved the overall rigidity and strength of the membrane material, resulting in an increase in tensile stress and Young’s modulus. This was because the appropriate degree of crosslinking did not excessively restrict the movement and deformation ability of the molecular chain segments, and the moderate N-acyl urea crosslinking point also promoted the orientation and arrangement of the molecular chain segments along the direction of stress, which helps to increase the fracture strain. However, when there are too many crosslinking points, the high rigidity will reduce the overall toughness of the material, which will lead to a decrease in the mechanical properties. This pattern of mechanical properties with changes in the amount of crosslinking was also observed when using the PCC-2 crosslinking agent (Figure 14). This may be attributed to the fact that both have the same functional group of carbodiimide crosslinking and have similar reaction mechanisms.

For the POC crosslinking agent (Figure 13b), with the increase in crosslinking agent content, the tensile strength increased significantly from 1.44 ± 0.08 MPa to 2.79 ± 0.14 MPa, Young’s modulus increased from 3.31 ± 0.73 MPa to 17.10 ± 3.97 MPa, and the elongation at break decreased from 296.0 ± 28.3% to 189.8 ± 6.6% when the concentration was increased from 3% to 20%. The imine group (-C=N-) in the POC crosslinking agent reacts with the carboxyl group to form a rigid ester bond (-C(O)-O-). The C=O and C-O bonds in the ester bond are covalent bonds with high polarity and high bond energy, thus showing high rigidity [34]. As the crosslinking reaction proceeds, the number of rigid crosslinking points increases, restricting the mobility of the polymer molecular segments. Therefore, the overall rigidity and tensile strength of the membranes were improved, reflected in the increase in tensile stress and Young’s modulus. However, the high density of rigid crosslinking points also impeded the relative sliding and stretching deformation of the molecular chain segments, leading to a decrease in the elongation at break.

For the PIC crosslinking agent (Figure 13c), the addition of the crosslinking agent decreased the tensile strength and Young’s modulus but increased the elongation at break compared with the sample without the crosslinking agent. The carboxyl and isocyanate groups in the waterborne PUSX undergo a crosslinking reaction to form the acetamide bond (-NH-CO-). The polarity of the acetamide bond is weak, and the rotational freedom of the bond is high. This flexibility in the local conformation allows the carbamate bond to undergo a certain degree of torsional deformation under external forces, thereby absorbing and dissipating energy, which improves the ductility and toughness of the material. As a result, the crosslinked fiber membrane could withstand larger strain deformation during tensile testing, but its tensile strength (tensile stress) was also correspondingly reduced. This flexible bonding structure also reduced the rigidity (Young’s modulus) of the material but improved the elongation at break capability.

## 4. Conclusions

In this study, we prepared waterborne silicone-modified polyurethane nanofibers for the first time, using waterborne electrospinning technology, with PEG as the template polymer, where three different functional crosslinking agents, namely carbodiimide (PCC), oxazoline (POC), and isocyanate (PIC), were incorporated in situ. The optimal crosslinking conditions for the three crosslinking agents were optimized and determined by systematically investigating the effects of crosslinking temperature and crosslinking time on the crosslinking reaction. Finally, the crosslinking conditions of a carbodiimide-based crosslinking agent (PCC-1), an oxazoline-based crosslinking agent (POC), and an isocyanate-based crosslinking agent (PIC) were determined to be 100 °C/30 min, 80 °C/24 h, and 100 °C/48 h, respectively. The morphology, structure, water resistance, and mechanical properties of different samples were characterized. The results showed that the addition of three crosslinking agents could maintain the fiber morphology after water immersion, and significantly improve the water resistance of the PUSX nanofiber membrane. The addition of the crosslinking agent had little effect on the water contact angle, and the main reason affecting the hydrophobicity was the presence of silicone. Different crosslinking agents had different effects on mechanical properties. The POC crosslinking agent can significantly improve tensile strength (when the content of the crosslinking agent was 20%, the tensile strength was 1.6 times that of the sample without the crosslinking agent) and Young’s modulus. The PIC crosslinking agent reduced the tensile strength and increased the elongation at break, that is, it enhanced the flexibility of the PUSX nanofiber membrane.

In short, we successfully constructed a highly water-resistant PUSX/PyC nanofiber membrane through green and environmentally friendly water-based electrospinning technology combined with simple heat treatment. The addition of different crosslinking agents can control the mechanical properties and swelling behavior, making it suitable for various fields, such as outdoor clothing, medical care, biomedicine, and oil–water separation.

## Figures and Tables

**Table 1:** Parameters of the crosslinking agent.

	PCC-1	POC	PIC	PCC-2
Appearance	White opaque	Light yellow liquid	Light yellow liquid	Light yellow liquid
Solid content (%)	40.0 ± 1.0	25.0 ± 1.5	70.0 ± 1.0	40.0 ± 1.0
Viscosity (mPa·s/20 °C)	Below 500	10–100	150–620	50–200
Ionicity	Nonionic	Nonionic	Nonionic	Nonionic
Functional group content	Carbodiimide (300 g·solid/eq)	Oxazoline (200 g·solid/eq)	NCO (8.7–9.7%)	Carbodiimide (600 g·solid/eq)
Solvent composition	Water	Water	Propylene glycol monomethyl ether acetate	Water

**Table 2:** Concentrations and conditions of crosslinking agents.

Crosslinking Agent	Content of Crosslinking Agent					Crosslinking Condition
PCC-1	3%	5%	10%	15%	20%	100 °C/30 min
POC	3%	5%	10%	15%	20%	80 °C/24 h
PIC	5%	10%	15%	20%	25%	100 °C/48 h
PCC-2	15%	30%	45%	60%	----	100 °C/30 min

## Data Availability

Data are contained within the article and Supplementary Materials.

## Supplementary Materials

The following supporting information can be downloaded at: https://www.mdpi.com/article/10.3390/polym16111500/s1, Figure S1: The synthesis of waterborne silicone-modified polyurethane; Figure S2: SEM images of PEG with molecular weight of 20,000 and PEG/PUSX ratios of 10/90 (a), 15/85 (b), 20/80 (c); Figure S3: SEM images of PEG with molecular weight of 100,000 and PEG/PUSX ratios of 10/90 (a), 15/85 (b), 20/80 (c) and 30/70 (d) and SEM images of 20/80 (c′) and 30/70 (d′) after water immersion; Figure S4: SEM images of PEG with a molecular weight of 500,000 and PEG/PUSX ratios of 5/95 (a), 10/90 (b), 15/85 (c) and 20/80 (d) and SEM images of 10/90 (b′), 15/85 (c′) and 20/80 (d′) after water immersion; Figure S5: SEM images of PU (a–d) and PUSX (e–h) nanofiber membranes at room temperature, 50 °C, 80 °C and 100 °C, heating for 24 h. The inset is a distribution of fiber diameters; Figure S6: The FTIR spectra of PUSX nanofibers under various crosslinking conditions with a crosslinking agent content of 15% (a) PCC-1, (b) POC, and (c) PIC; Figure S7: SEM images of PUSX nanofibers before (a_1_–d_1_) and after (a_2_–d_2_) water immersion at 15% content of PCC-2 with different crosslinking conditions. The crosslinking conditions were (a) room temperature, (b) 80 °C/24 h, (c) 100 °C/24 h and (d) 100 °C/30 min. (e) Average diameter and diameter change rate, (f) weight and area change rate, (g) water retention, and (h) water contact angle of PUSX nanofibers at 15% PCC-2 content; Figure S8: The porosity of POC 15% content with different crosslinking conditions; Figure S9: SEM images of PUSX nanofibers with PCC-2 crosslinking agent before (a_1_–d_1_) and after (a_2_–d_2_) immersion in water at different contents. (e) Average diameter, (f) weight and area change rate, (g) water retention rate, and (h) water contact angle of the PUSX nanofibers with PCC-2 crosslinking agent; Figure S10: The FTIR spectra of PCC-1 and PCC-2 crosslinking agents; Figure S11: SEM images of PEG nanofiber membranes before (a_1_–c_1_) and after (a_2_–c_2_) water immersion with different crosslinking agents (a) PCC-1, (b) POC, and (c) PIC; Figure S12: Stress-strain curve of PUSX nanofiber membrane without crosslinking agent.
