# Supplementary material for: Preparation of Waterborne Silicone-Modified Polyurethane Nanofibers and the Effect of Crosslinking Agents on Physical Properties
Source: Polymers (Basel). 2024 May 24;16(11):1500. doi: 10.3390/polym16111500 (PMC11174862; doi:10.3390/polym16111500)
Supplement: Supplementary file 1 [file polymers-16-01500-s001.zip › polymers-2997790-supplementary.pdf]

polymers

MDPI

Article

Preparation of waterborne silicone-modified polyurethane nanofibers and the effect of crosslinking agents on physical properties

Fang Li [1], Kai Weng [1], Asumi Nakamura [1], Keishiro Ono [1], Toshihisa Tanaka [1,*], Daisuke Noda [2], Masaki Tanaka [2], Shinji Irifune [2] and Hiromasa Sato [3]

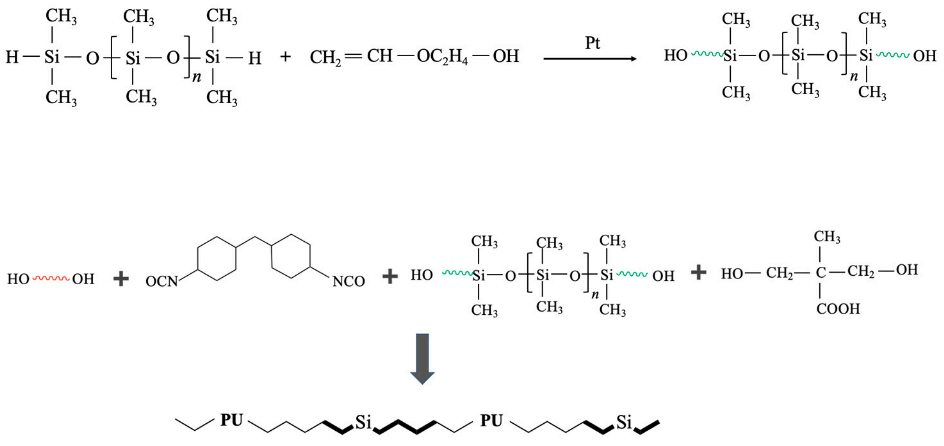

(1-1)

(1-2)

Figure S1. The synthesis of waterborne silicone-modified polyurethane.

Supplementary Discussion 1: Effect of molecular weight and content of polyethylene glycol (PEG) on PUSX nanofibers

In this study, PEG with molecular weights of 20,000, 100,000 and 500,000 was used as a template polymer in an attempt to achieve fiberization of PUSX. We selected different ratios of PEG to PUSX (5/95, 10/90, 15/85, 20/80 and 30/70) to prepare PUSX/PEG spinning solution. The results showed that PEG (20,000) could not achieve the fiberization of PUSX (Figure S2). When PEG (100,000)/PUSX was 20/80 and 30/70, the fiberization of PUSX could be achieved (Figure S3 c, d). However, the fiber morphology after immersion in water cannot be maintained (Figure S3 c′, d′). This is due to the high content of PEG dissolved in water. Figure S4 shows SEM images of PEG with a molecular weight of 500,000 and different PUSX ratios. When PEG/PUSX was 10/90 (Figure S4 b), fiberization of PUSX could be achieved. PEG molecular chains of high molecular weight are longer, and more entanglements between molecules increase the viscosity of solution, which leads to the formation of fibers under the action of electric field force. When PEG/PUSX was 15/85 and 20/80 (Figure S4 c, d), the fiberization of PUSX was also achieved. However, because PEG is easily soluble in water, it is difficult to maintain the morphology of the fibers (Figure S4 c′, d′) after water immersion. These results indicated that PEG with a molecular weight of 20,000 cannot achieve the fiberization of PUSX, and PEG with a molecular weight of 100,000 required a 20wt% addition to achieve it. Considering the solubility of PEG in water, we chose high molecular weight PEG (500,000) with a PEG/PUSX ratio of 10/90 for subsequent studies, aiming to achieve fiberization while maintaining fiber morphology as much as possible. Therefore, this paper selected the PEG/PUSX ratio of 10/90 for subsequent research.

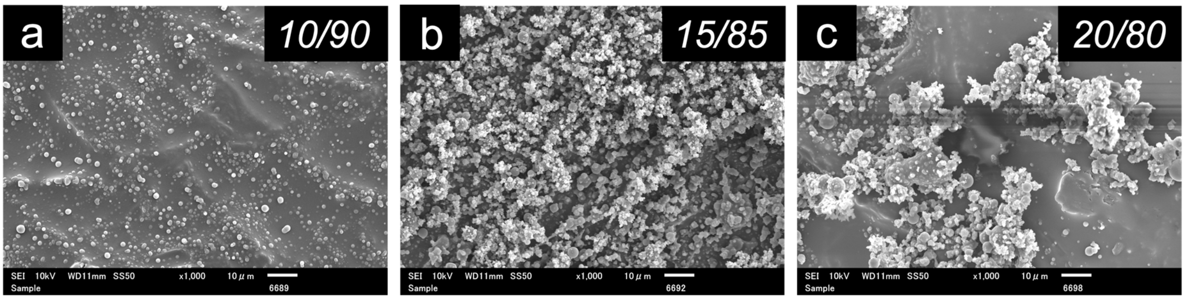

Figure S2. SEM images of PEG with molecular weight of 20,000 and PEG/PUSX ratios of 10/90 (**a**), 15/85 (**b**), 20/80 (**c**).

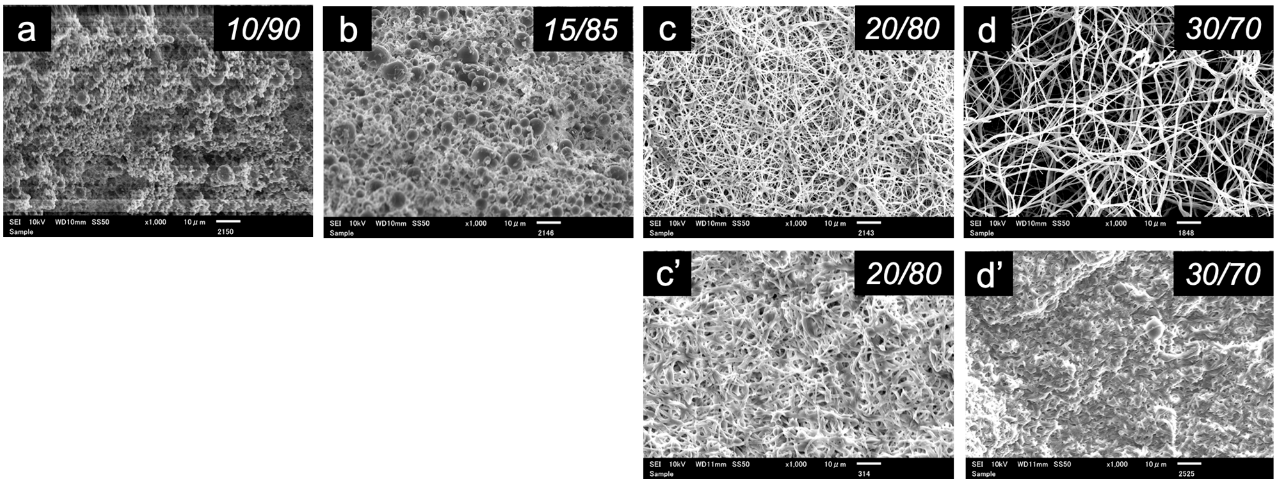

Figure S3. SEM images of PEG with molecular weight of 100,000 and PEG/PUSX ratios of 10/90 (**a**), 15/85 (**b**), 20/80 (**c**) and 30/70 (**d**) and SEM images of 20/80 (**c**′) and 30/70 (**d**′) after water immersion.

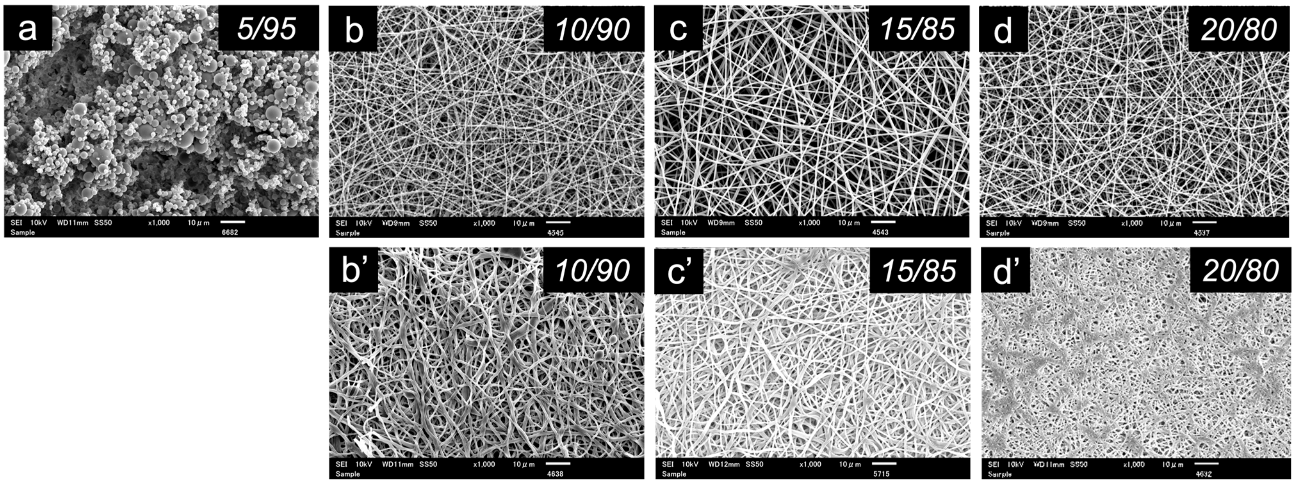

Figure S4. SEM images of PEG with a molecular weight of 500,000 and PEG/PUSX ratios of 5/95 (**a**), 10/90 (**b**), 15/85 (**c**) and 20/80 (**d**) and SEM images of 10/90 (**b**′), 15/85 (**c**′) and 20/80 (**d**′) after water immersion.

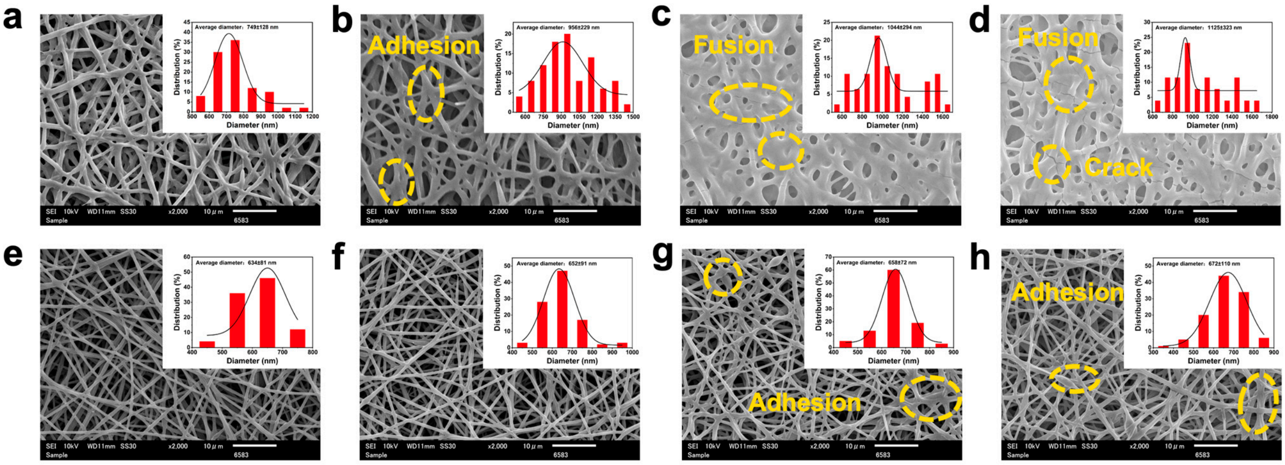

Figure S5. SEM images of PU (**a**-**d**) and PUSX (**e**-**h**) nanofiber membranes at room temperature, 50°C, 80°C and 100°C, heating for 24 h. The inset is a distribution of fiber diameters.

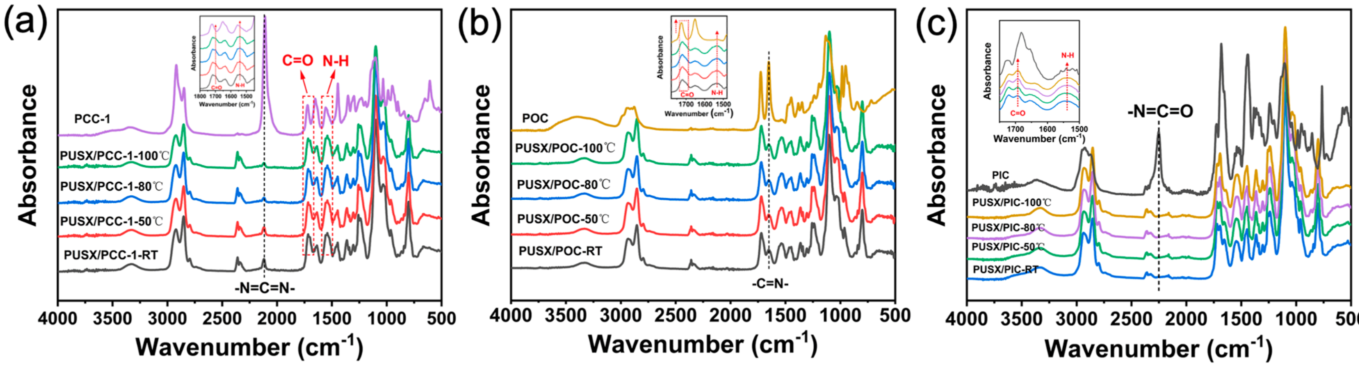

Figure S6. The FTIR spectra of PUSX nanofibers under various crosslinking conditions with a crosslinking agent content of 15% (**a**) PCC-1, (**b**) POC, and (**c**) PIC.

Supplementary Discussion 2:

Figure S7 (a-d) shows the fiber morphological changes of PUSX nanofibers before and after water immersion after being treated with different crosslinking conditions using a 15% PCC-2 crosslinking agent. Immersion in water can cause the fibers to swell and increase in diameter, but compared to the room temperature crosslinked sample, the rate of diameter changes showed a little increase (Figure S7e). As shown in Figure S7f, different crosslinking conditions had little effect on the rate of change in weight and area of nanofiber membranes before and after water immersion. Similarly, crosslinking conditions had almost no effect on the water retention and hydrophobicity by water contact angle of the fiber membrane (Figure S7g and h). Considering that PCC-2 and PCC-1 contain the same functional groups (-N=C=N-), it is expected that their reaction activities are similar. Based on the previous optimization results of PCC-1, we have comprehensively selected 100°C/30 min as the final crosslinking condition for PCC-2.

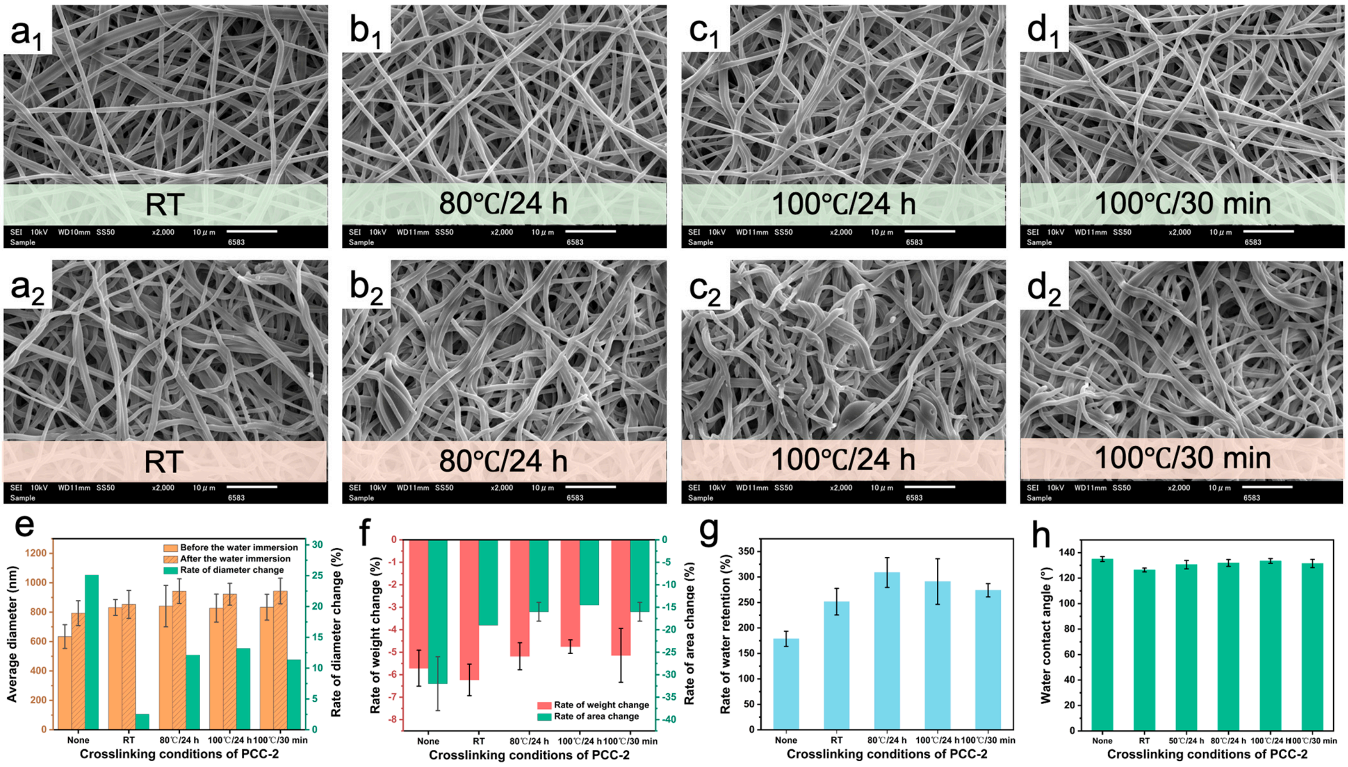

Figure S7. SEM images of PUSX nanofibers before (**a**$_1$-**d**$_1$) and after (**a**$_2$-**d**$_2$) water immersion at 15% content of PCC-2 with different crosslinking conditions. The crosslinking conditions were (**a**) room temperature, (**b**) 80℃/24 h, (**c**) 100℃/24 h and (**d**) 100℃/30 min. (**e**) Average diameter and diameter change rate, (**f**) weight and area change rate, (**g**) water retention, and (**h**) water contact angle of PUSX nanofibers at 15% PCC-2 content.

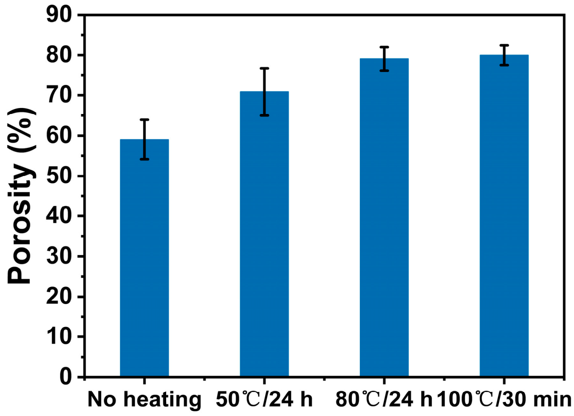

Figure S8. The porosity of POC 15% content with different crosslinking conditions.

Supplementary Discussion 3:

Figure S9(a-d) demonstrates the morphological changes of PUSX nanofibers before and after water immersion with the addition of different contents of PCC-2 crosslinking agent. The addition of crosslinking agent showed the morphological stability of the fibers to a certain extent. However, compared with other crosslinking agents such as PCC-1, POC and PIC, the PCC-2 crosslinking agent imparts poor water resistance to the nanofiber membranes. As shown in Figure S9e and **f**, the average diameter, weight, and area of the fibers showed larger rates of change after water immersion. Although PCC-1 and PCC-2 have the same crosslinking functional groups, the relatively weaker crosslinking effect of

PCC-2 may be attributed to several factors. On the one hand, the two crosslinking agents have different functional group contents (Table 1) and slight differences in their chemical structures (Figure S10). This may lead to variations in the quantity or nature of the crosslinking points, thereby affecting the overall crosslinking efficiency. On the other hand, there might be phase separation between the PCC-2 crosslinking agent and PUSX. The high addition of crosslinking agents leads to a significant increase in their proportion in the PUSX matrix. This could lead to enhanced interaction forces among the crosslinking agents themselves, thereby resulting in phase separation from PUSX.

Figure S9g and h show the water retention and water contact angle of nanofiber membranes with different PCC-2 contents. The data showed that the content of PCC-2 crosslinking agent had no significant effect on these two indexes.

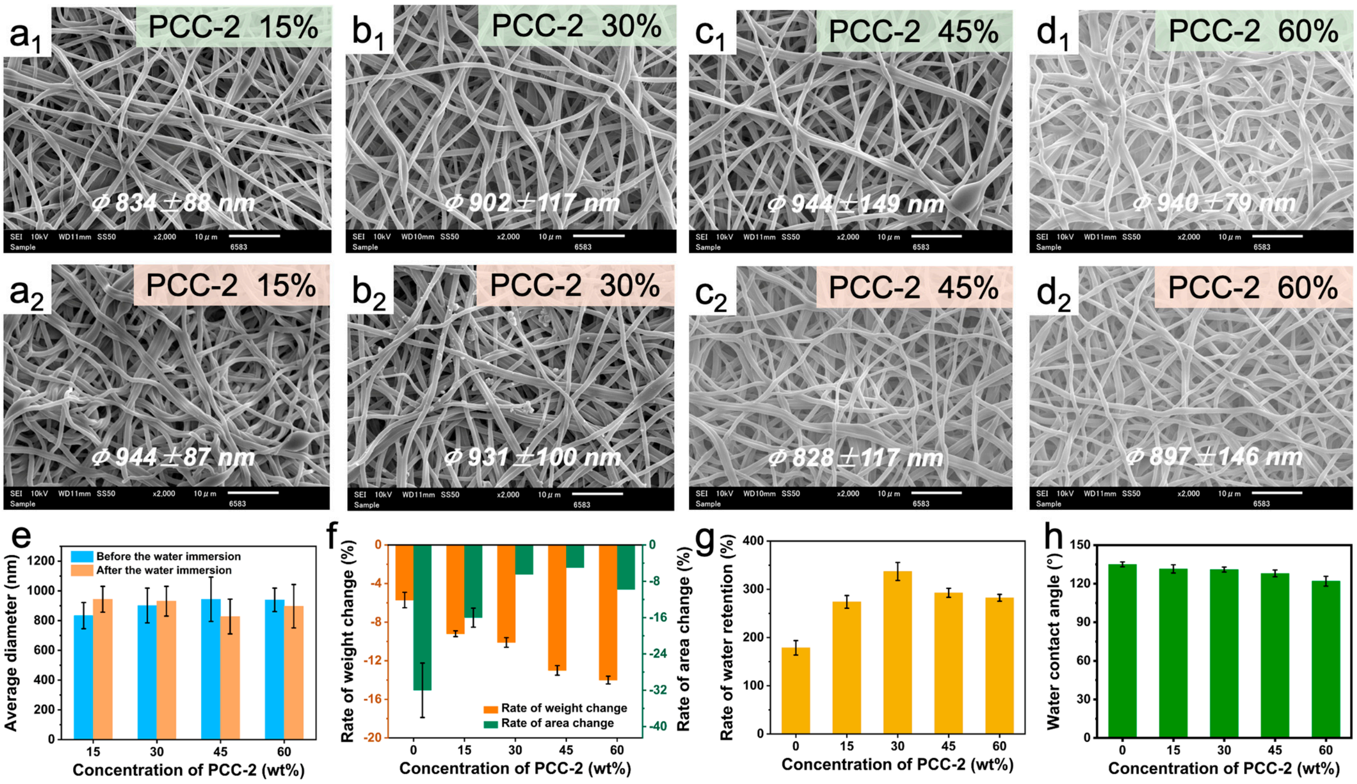

Figure S9. SEM images of PUSX nanofibers with PCC-2 crosslinking agent before (**a**1-**d**1) and after (**a**2-**d**2) immersion in water at different contents. (**e**) Average diameter, (**f**) weight and area change rate, (**g**) water retention rate, and (**h**) water contact angle of the PUSX nanofibers with PCC-2 crosslinking agent.

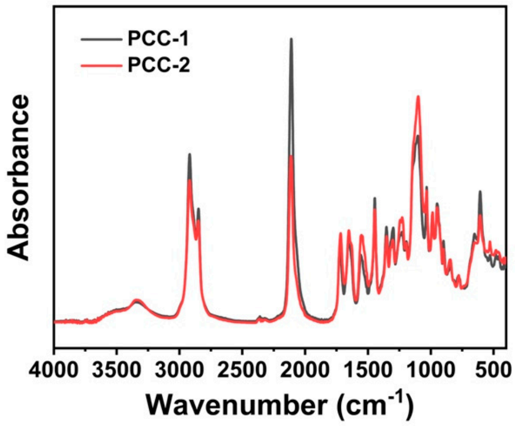

Figure S10. The FTIR spectra of PCC-1 and PCC-2 crosslinking agents.

Supplementary Discussion 4: Experimental validation of the reaction between PEG and crosslinking agent

Considering the terminal hydroxyl groups of PEG may react with the crosslinking agents, we conducted the following experiments to verify whether PEG undergoes crosslinking reactions with three different functional group crosslinking agents. Firstly, we prepared a 5 wt% PEG spinning solution as a control sample in distilled water. Additionally, 5 wt% of the three crosslinking agents relative to PEG content were added to the spinning solution. Using an electrospinning device, pure PEG nanofibers (not shown in this work), PEG/PCC-1, PEG/POC, and PEG/PIC nanofiber membranes were obtained at a voltage of 15 kV. Considering the dissolution of PEG at high temperatures, we chose to heat the samples at 50°C for 48 h to promote the crosslinking reaction. As shown in Figure S11 (a_1-c_1), the different crosslinking agents did not affect the fiber formation of the PEG nanofibers. To verify whether PEG participated in the crosslinking reaction, the prepared crosslinked membranes were immersed in a dish containing distilled water for 3 hours and then dried in an oven. Figure S11 (a_2-c_2) shows the SEM images after water immersion. The nanofiber membranes with added PCC-1 and PIC crosslinking agents maintained their fibrous morphology, although the fibers were swollen. However, the sample with the added POC crosslinking agent showed a completely melted morphology when collected at the bottom of the dish (Figure S11b_2) showed a completely melted morphology. This result indicates that PEG cannot react with the POC crosslinking agent. Therefore, in the long-term water immersion test (24 h), the increased water retention of the nanofiber membranes was due to the swelling of uncrosslinked PEG, as shown in Figure S11 b_2.

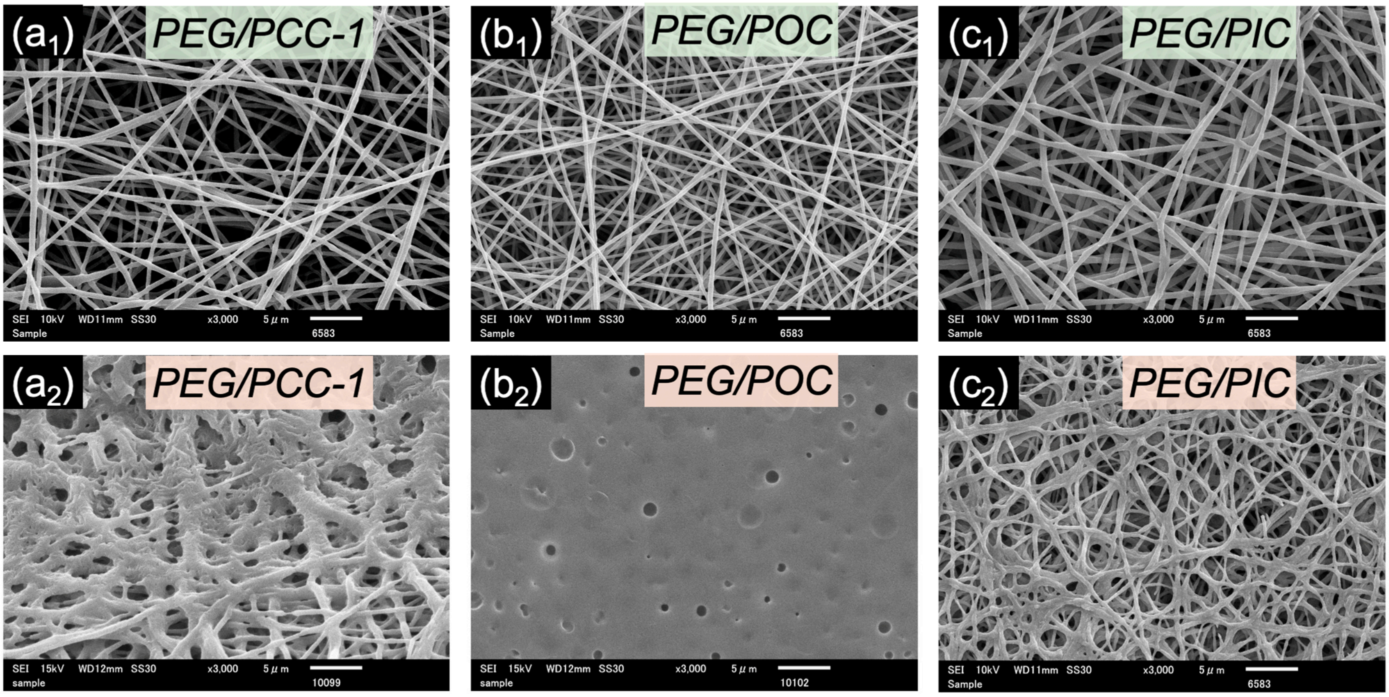

Figure S11. SEM images of PEG nanofiber membranes before (**a**1-**c**1) and after (**a**2-**c**2) water immersion with different crosslinking agents (**a**) PCC-1, (**b**) POC, and (**c**) PIC.

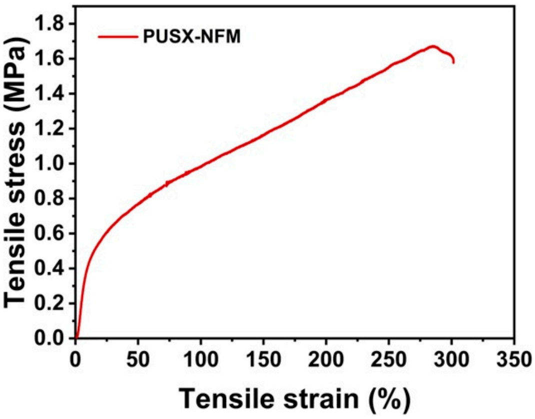

Figure S12. Stress-strain curve of PUSX nanofiber membrane without crosslinking agent.
